# Peripartum Cardiomyopathy in a Multigravida Patient

**DOI:** 10.7759/cureus.36492

**Published:** 2023-03-21

**Authors:** Mitchell Cross, Joel H Schirding, Kristina Domanski

**Affiliations:** 1 Emergency Medicine, University Medical Center, Las Vegas, USA

**Keywords:** multigravida, general obgyn, peripartum dilated cardiomyopathy, acute decompensated systolic heart failure, emergency medicine resuscitation, ppcm, peripartum cardiomypathy

## Abstract

Peripartum cardiomyopathy (PPCM) is a potentially life-threatening pregnancy-associated disease that typically arises in the third trimester or up to six months postpartum. This case report focuses on a multigravida patient that has been pregnant a total of 12 times. The patient had no known past medical history apart from pre-eclampsia. Information related to this disease is scarce, and its pathophysiology remains poorly understood. This case report will further enhance scientific as well as medical literature by improving healthcare providers' knowledge and understanding of PPCM, which will ultimately improve patient outcomes through swift recognition and early treatment initiation.

## Introduction

Peripartum cardiomyopathy (PPCM) is a rare complication that occurs in approximately 1 in 4,000 pregnancies in the US each year [1]. It typically occurs in the later stages of pregnancy or up to six months postpartum and is associated with high morbidity and mortality [2]. The pathophysiology of this disease is not entirely understood; however, it is postulated to be a direct result of hormonal imbalances and physiologic changes during and post-pregnancy that stress the cardiovascular system beyond its normal capabilities. A guideline for the noninvasive characterization of this disease process was first developed in 1999 through improvements in echocardiography, with an agreed-upon criterion that the patient's ejection fraction must be <45%. The European Society of Cardiology has also created a standardized, working definition of this disease using the following inclusion criteria: reduced ejection fraction of less than 45% that develops towards the end of pregnancy or six months postpartum in a patient with no known history of structural heart disease [3]. The presentation of this disease process is similar to that of those patients suffering from dilated cardiomyopathy and the definitive diagnosis is made through echocardiography. 

Individuals with this disease typically present to the emergency department and require rapid evaluation, treatment, and specialist consultation. The morbidity and mortality of this disease process can be as high as 50%, which highlights the need for prompt stabilization and resuscitation of this patient population [4]. Understanding the typical presentation will help the emergency department physician efficiently care for these patients and hopefully improve clinical outcomes.

## Case presentation

A 38-year-old G12P11 female presented to the emergency department by emergency medical services (EMS) 11 days postpartum from an uncomplicated, spontaneous vaginal delivery. The patient reported shortness of breath, an inability to tolerate lying flat at night, as well as increased swelling of her bilateral lower extremities. She denied any chest pain, hemoptysis, recent travel, surgeries, or personal history of pulmonary embolism. Aside from multiparity and the history of preeclampsia, the patient denied any other significant past medical history. She reported regular tobacco use of approximately 1/4 packs per day but denied any illicit drug or alcohol use. The patient had poor prenatal care, and medical follow-up secondary to financial constraints. 

On evaluation, the patient was tachypneic and uncomfortable with four-word dyspnea. Initial vital signs were significant for an oxygen saturation of 90% and tachycardia to the 110s, but her blood pressure was stable. She was placed on 4L of supplemental oxygen via nasal cannula and responded appropriately to this treatment intervention with a saturation of 98%. Lung auscultation revealed bibasilar crackles and bedside pulmonary ultrasound was significant for B-lines consistent with pulmonary edema.

Preliminary cardiac ultrasound performed at the bedside was significant for a moderately reduced ejection fraction, which was estimated to be less than 40% (Video 1). During direct visualization of the parasternal long axis view, the left ventricle had poor contractility consistent with findings of heart failure. 

**Video 1:** Parasternal long axis cardiac ultrasound

Diuresis with 40mg of furosemide was initiated due to clinical concerns for volume overload. Laboratory studies are depicted in Table 1. Aside from anemia and a mildly elevated brain natriuretic peptide (BNP) and troponins, all other labs were within normal limits. The patient's EKG was significant for sinus tachycardia with a heart rate of 129 bpm with PR, QRS, and QTc intervals within normal limits and no evidence of ST segment elevations, depressions, or other acute findings of myocardial ischemia.

**Table 1:** Emergency department laboratory results

Lab	Value	Reference Range
Brain natriuretic peptide	1,422 pg/ml	< 100 pg/ml
Troponin, initial	74 ng/L	< 14 ng/L
Troponin, repeat	84 ng/L	< 14 ng/L
Lactate	2.12 mmol/L	0.5-1.90 mmol/L
White blood cell	10,140 K/mm3	3.10-10.2 K/mm3
Hemoglobin	11.5 g/dL	13.1-16.8 g/dL
Platelets	253,000 K/mm3	119,000-332,000 K/mm3
Sodium	136 mmol/L	136-145 mmol/L
Potassium	3.9 mmol/L	3.5-5.1 mmol/L
Creatinine	.73 mg/dL	.55-1.30 mg/dL

The patient's chest X-ray was significant for diffuse pulmonary infiltrates versus edema and revealed a stable cardiomediastinal silhouette (Figure 2). Given the concern for pulmonary embolism due to this patient's shortness of breath and hypoxia, a CT angiogram of her chest was obtained. This imaging study revealed diffuse bilateral pulmonary infiltrates with small bilateral pleural effusions but no evidence of pulmonary embolus (Figure 3). During this patient's hospitalization a formal transthoracic echocardiogram was also obtained that was significant for left ventricular systolic function being severely reduced with a reported ejection fraction of 30%-35%. The mitral valve leaflets appeared thickened but opened well with moderate mitral regurgitation. Additionally, the right ventricle appeared to be moderately dilated with moderate to severe tricuspid insufficiency. There was noted to be an elevated right ventricular systolic pressure of 50-60 mmHg. There was no evidence of aortic valve stenosis or pericardial effusion. 

**Figure 1:**
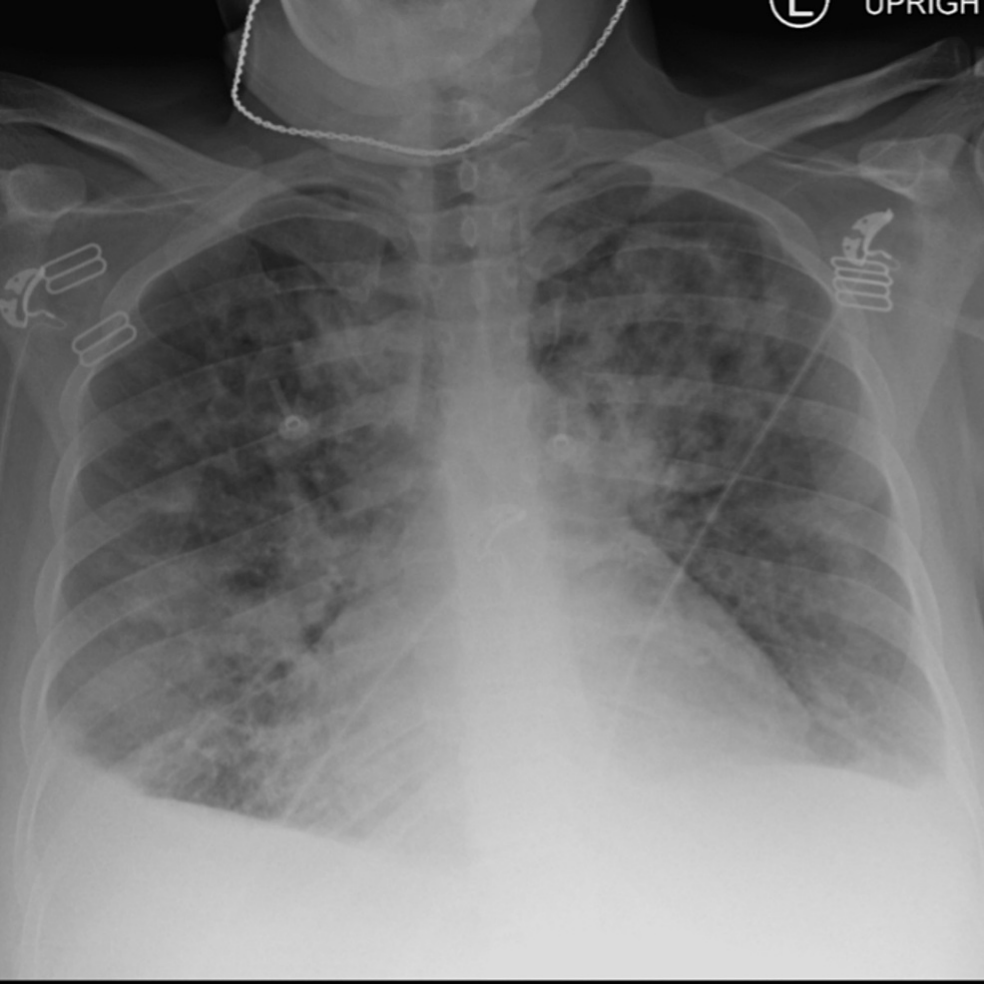
Chest X-ray Diffuse pulmonary vascular congestion was present with no evidence of pneumothorax.

**Figure 2:**
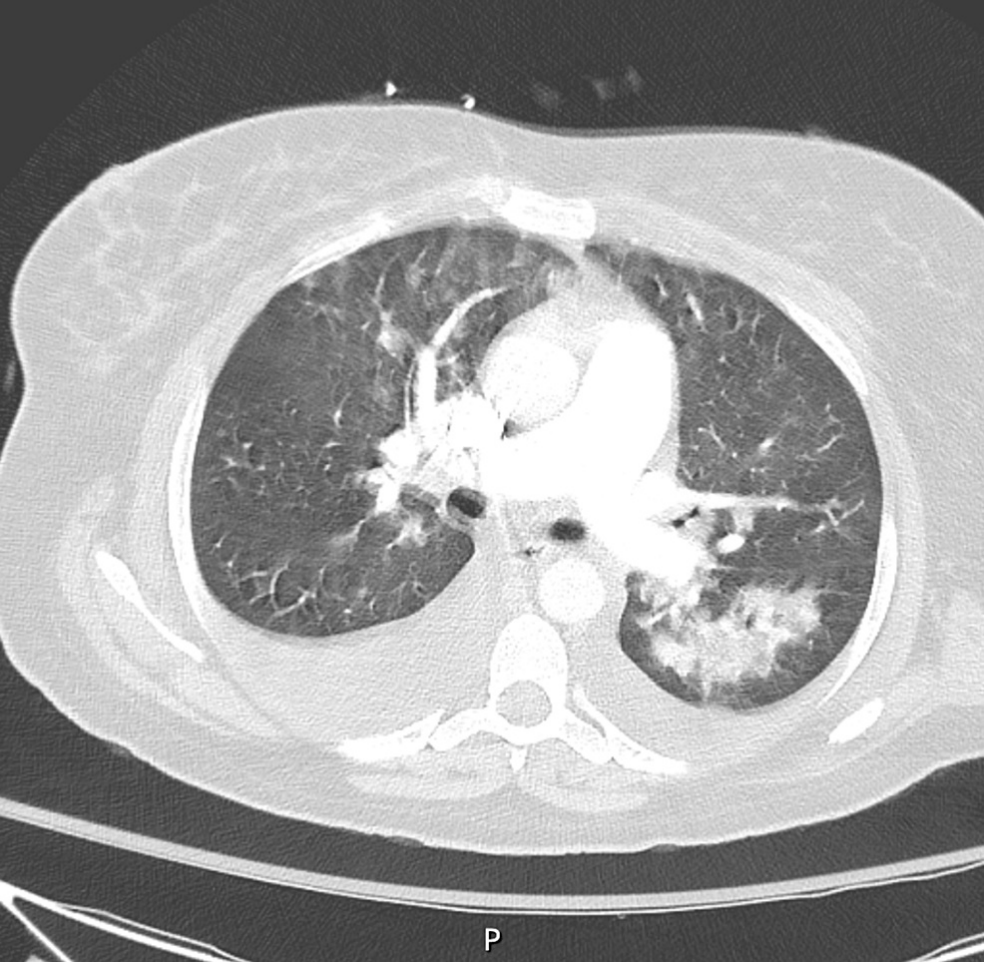
CT angiogram chest Bifurcation of pulmonary artery

The obstetrics and gynecology (OB/GYN) team evaluated the patient and recommended no acute surgical interventions, and deferred to cardiology for PPCM management. The cardiology service recommended diuresis and the initiation of goal-directed medical therapy in the setting of decompensated heart failure. The patient was started on furosemide 80mg twice daily as well as lisinopril 20mg daily. Beta-blockers were also recommended to be administered once the patient's hemodynamic status had been approved and she was no longer in a decompensated state of heart failure. The patient was discharged three days after she had initially presented to the emergency department. Goal-directed medical therapy was initiated during her hospital course, and she was discharged with carvedilol, furosemide, and lisinopril. She was also given outpatient cardiology, and OB/GYN follow-up but never attended these appointments. 

## Discussion

Peripartum cardiomyopathy frequently arises in multi-gestational patients. The etiology of PPCM is unclear, but there have been several hypotheses proposed to explain the etiology and pathogenesis of this disease process, including viral infections, myocarditis, autoimmune factors, inflammatory cytokines, and abnormal hemodynamic responses to physiological changes in pregnancy that may potentially play a role in the development of peripartum cardiomyopathy [1]. Female physiology changes significantly during pregnancy, and the hemodynamic changes that occur are thought to contribute to this disease. Preload increases secondary to the increase in blood volume and red cell mass, which ultimately increases cardiac output by nearly 20-30% during the second and third trimesters. These changes, when combined with other hormonal changes in pregnancy, can alter the sarcomere protein responsible for myocardial contractility and ultimately affect ejection fraction [5]. Additionally, there have been many risk factors implicated in the development of this disease process. The incidence of PPCM is strongly associated with age, as half of all reported cases are in women over the age of 30 [1]. Additional risk factors include a history of preeclampsia, hypertension, African American race, as well as a history of multiple gestations. The patient presented in this case report had been pregnant a total of 12 times which ultimately exceeded her heart's ability to withstand all the physiologic and hormonal changes associated with pregnancy. While the development of PPCM has high morbidity and mortality, it does have the possibility of full recovery. In 2017, pregnancy-related mortality by all causes was 17.3% for every 100,000 live births (17,300 deaths), and of that, 11.5% (1,990 deaths) were due to PPCM [6]. The overall prognosis for this disease largely depends on the patient's ejection fraction at the time of onset. About 50-70% of patients have a gradual improvement in ventricular function by six months; however, embolic events are a leading cause of mortality, with a rate estimated to be near 30% [7].

## Conclusions

Individuals presenting to the emergency department during late antepartum or postpartum periods with signs and symptoms consistent with heart failure exacerbation require swift intervention and stabilization to prevent rapid clinical deterioration. Peripartum cardiomyopathy is a rare but potentially deadly variant of heart failure that is multifactorial in etiology and typically occurs in later pregnancy stages as well as the postpartum period. PPCM manifests primarily as ventricular systolic dysfunction from a global reduction in myocardial contraction, causing a reduced ejection fraction. It is theorized that PPCM could be mediated through many mechanisms, including immunological factors, an abnormal hormone response, abnormal inflammatory changes, or myocarditis. Early diagnostic testing is essential to the appropriate initiation of treatment interventions to improve the morbidity and mortality associated with this disease process. Modalities such as echocardiography revealing evidence of left ventricular systolic dysfunction are crucial in determining the diagnosis.

Women with a small decrease in ejection fraction from their normal baseline typically have a good prognosis, but those with significant declines in ejection fraction ultimately have a high risk of decompensation and death. Although there is no exact data amount specified in the literature, the recurrence rate of PPCM is high in subsequent pregnancies. Therefore, women should be monitored closely by a multidisciplinary healthcare team in the event that they become pregnant again. Emergency medicine clinicians must be educated in disease recognition, management, and awareness of potential complications in order to provide effective, high-quality care to reduce maternal morbidity and mortality associated with peripartum cardiomyopathy. 
